# Immobilization of Multi-Enzymes on Support Materials for Efficient Biocatalysis

**DOI:** 10.3389/fbioe.2020.00660

**Published:** 2020-06-30

**Authors:** Kongliang Xu, Xuexiao Chen, Renchao Zheng, Yuguo Zheng

**Affiliations:** ^1^Key Laboratory of Bioorganic Synthesis of Zhejiang Province, College of Biotechnology and Bioengineering, Zhejiang University of Technology, Hangzhou, China; ^2^Engineering Research Center of Bioconversion and Biopurification of Ministry of Education, Zhejiang University of Technology, Hangzhou, China

**Keywords:** multi-enzyme immobilization, co-immobilization, support materials, immobilization technologies, biocatalysis

## Abstract

Multi-enzyme biocatalysis is an important technology to produce many valuable chemicals in the industry. Different strategies for the construction of multi-enzyme systems have been reported. In particular, immobilization of multi-enzymes on the support materials has been proved to be one of the most efficient approaches, which can increase the enzymatic activity via substrate channeling and improve the stability and reusability of enzymes. A general overview of the characteristics of support materials and their corresponding attachment techniques used for multi-enzyme immobilization will be provided here. This review will focus on the materials-based techniques for multi-enzyme immobilization, which aims to present the recent advances and future prospects in the area of multi-enzyme biocatalysis based on support immobilization.

## Introduction

In nature, almost all the cascade reactions in cell are catalyzed by the cooperation of various enzymes (Ricca et al., 2011; Sheldon and Woodley, 2018; Shi et al., 2018). For example, in the tricarboxylic acid (TCA) cycle, malate dehydrogenase (MD), citrate synthase (CS), and other enzymes regulate metabolic biosynthetic pathways by controlling the formation of intermediates (Barnes and Weitzman, 1986). Multi-enzyme biocatalysis is an important technology to produce many valuable chemicals in the industry (Britton et al., 2018; Huffman et al., 2019), which integrates several biocatalytic transformations, bridging the gap between single-enzyme catalysis, and whole-cell catalysis. Inspired from multi-enzyme reactions *in vivo*, researchers have attempted to construct functional multi-enzyme systems *in vitro* to produce desired chemicals (Mayer et al., 2001; Yang et al., 2019). Different strategies for the construction of multi-enzyme systems have been reported, including fusion of enzymes, enzyme-scaffold complexes and co-immobilization (Hwang and Lee, 2019).

Multi-enzyme immobilization is a technology that co-localizes multiple enzymes on suitable supports/carriers or combines enzymes using a linker without supports (Ren et al., 2019). Enzymes will be close to one another and the mass transfer limitation can be reduced through co-immobilization, which has been proved to increase the enzymatic activity via substrate channeling and improve the stability and reusability. As support materials can strongly affect the properties of enzymes, support selection has been considered as a hot topic in the field of enzyme immobilization. To date, various materials, such as graphene, carbon nanotubes (CNTs), metal-organic frameworks (MOFs), DNA nanostructures, polymers and silica, have been applied for multi-enzyme immobilization, which can efficiently protect enzymes from heavy metals, high temperatures, and other biologically challenging conditions (Sheldon and Woodley, 2018; Ren et al., 2019).

**Figure 1:**
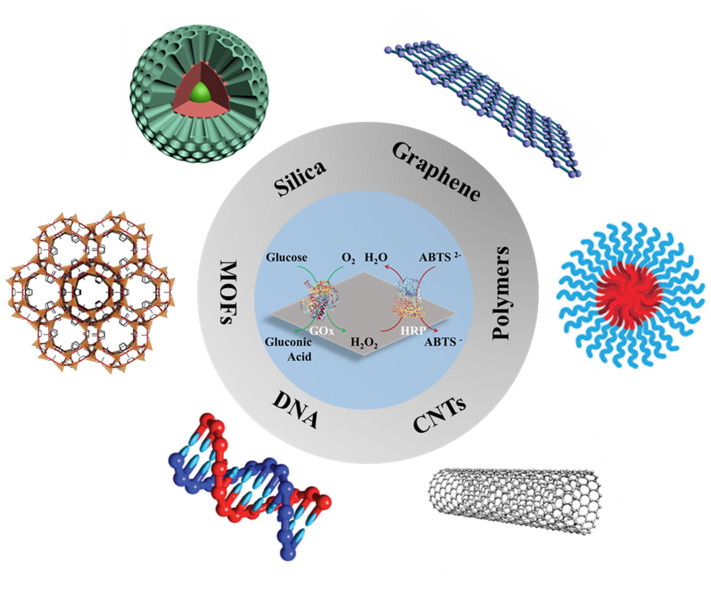
Schematic images of various materials for multi-enzyme immobilization. Reproduced with permissions from ACS Publications, John Wiley and Sons, and The Royal Society of Chemistry.

This review will provide a general overview of the characteristics of support materials and their corresponding attachment techniques used for multi-enzyme immobilization. Although some recent reviews have already summarized the co-immobilized techniques and their corresponding applications (Ansari and Husain, 2012; Shi et al., 2018; Hwang and Lee, 2019; Ren et al., 2019; Giannakopoulou et al., 2020), the review emphasizing on the supports is rare to date (Jia et al., 2014). This review will focus on the recent support materials for multi-enzyme immobilization (Figure 1), which aims to present the recent advances and future prospects in the area of multi-enzyme biocatalysis.

## Multi-Enzyme Immobilization Technologies

**Figure 2:**
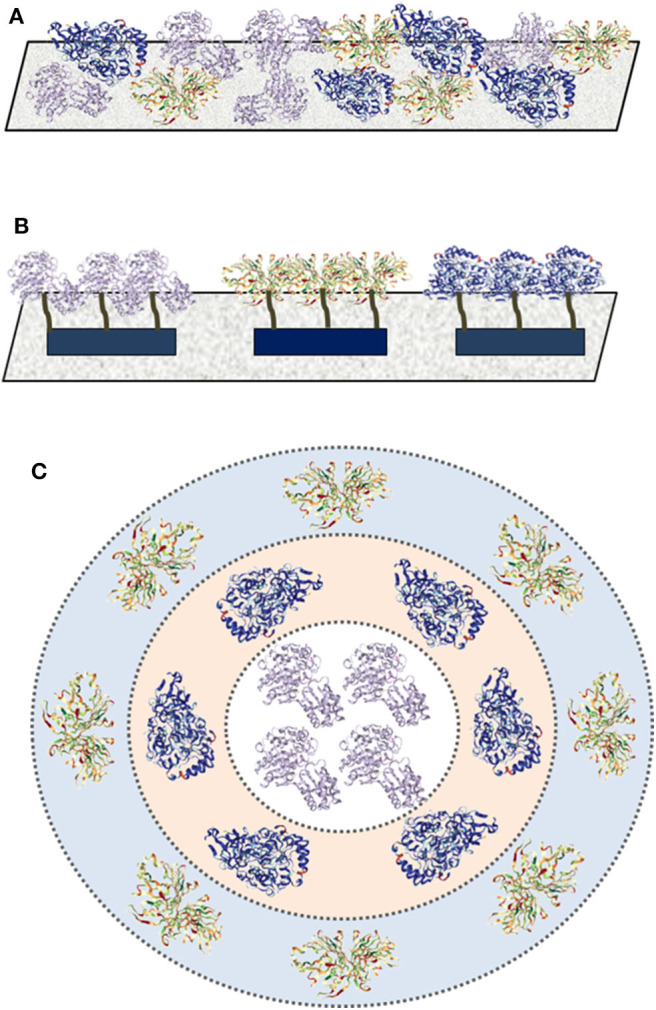
Three techniques for multi-enzyme immobilization. **(A)** Random co-immobilization; **(B)** positional co-immobilization; and **(C)** compartmentalization.

There are three main techniques for multi-enzyme immobilization, including random co-immobilization, positional co-immobilization, and compartmentalization (Figure 2; Hwang and Lee, 2019). As the basic interaction between enzymes and supports is critical to understand the co-immobilized techniques, basic types of immobilization for enzymes will be discussed briefly before the introduction of the three techniques.

### Basic Types of Immobilization

Methods for enzyme immobilization can be divided into three categories, including binding to a support, cross-linking, and encapsulation (entrapment) (Mosbach and Mattiasson, 1976; Sheldon and van Pelt, 2013). (a) Binding to a support refers to immobilize enzyme on suitable carriers by hydrophobic action, ion interaction, hydrogen bonding, specific affinity, covalent bonding, etc. (b) Cross-linking is a support-free immobilization method, which can bind enzymes together to form a network by a cross-linker. For example, the Schiff base bond can be formed between the double aldehyde group of glutaraldehyde (a typical cross-linking agent) and the amino group of the enzyme molecules, which can efficiently connect enzymes together. (c) Encapsulation means the entrapment of enzymes in a network or a hollow fiber or a microcapsule during material preparation.

### Random Co-immobilization

Random co-immobilization is the simplest strategy to create a multi-enzyme system, in which multiple enzymes are randomly attached on the surface or embedded inside the carrier via adsorption, encapsulation, covalent attachment, cross-linking, etc. (Figure 2A). For instance, a tri-enzyme biocatalyst for one-pot starch hydrolysis was prepared by ammonium sulfate aggregation and followed by glutaraldehyde-assisted cross-linking, in which alpha amylase, glucoamylase, and pullulanase were randomly co-immobilized as cross-linked enzyme aggregates (Talekar et al., 2013). The resultant hydrolytic activity was well-maintained up to five cycles without obvious changes and the thermal stability was improved effectively.

### Positional Co-immobilization

Positional co-immobilization has been proved to be an effective strategy for ordered multi-enzyme immobilization, which can control and improve the cascade enzymatic reaction rates via adjusting the immobilized sequence (Figure 2B). Polymers and DNA nanostructures are the common-used carriers for positional co-immobilization for their ability to control the relative positions of enzymes via specific interactions (see sections DNA Nanostructures and Polymers parts for more discussions).

### Compartmentalization

As a mimic of natural enzyme organization in cellular environments, compartmentalization can spatially separate enzymes with different patterns and ratios (Figure 2C), which can protect enzymes against proteolysis, microbial degradation, or other harmful environments (Marguet et al., 2013). For example, Ge and Liu's group constructed a compartmentalized multi-enzyme system based on inorganic nanocrystal-protein complexes via a simple precipitation method (Li et al., 2014), which exhibited enhanced overall catalytic performance compared with free enzymes. Horseradish peroxidase (HRP) was first mixed with CuSO_4_ in water to form the HRP-incorporated complexes, then glucose oxidase (GOx) was adsorbed on the surface of the complexes by the coordination interaction between Cu^2+^ and amino acids of protein. Polymersome and colloidosome are the common materials for compartmentalized multi-enzyme immobilization (see sections Polymers and Silica parts for more discussions).

## Support Materials for Multi-Enzyme Immobilization

A great number of materials with different shapes/sizes, porous/non-porous structures, and binding functionalities have been designed as carriers for multi-enzyme immobilization and the following 6 main catalogs of support materials (Figure 1) will be overviewed.

### Graphene and Its Derivatives

Graphene is a flat single-layer two-dimensional carbon atoms tightly packed into honeycomb lattice (Geim and Novoselov, 2007). Since its first isolation in 2004 (Novoselov et al., 2004), graphene has attracted tremendous scientific interests owing to its extraordinary properties, such as large theoretical specific surface area (2,630 m^2^ g^−1^), high intrinsic mobility (200,000 cm^2^ v^−1^ s^−1^), high Young's modulus (~1.0 TPa) and high thermal conductivity (~5,000 W m^−1^ K^−1^) (Zhu et al., 2010). There are four primary ways to produce graphene, including physical exfoliation, epitaxial growth, chemical vapor deposition, and oxidation-reduction method (Zhu et al., 2010). In particular, graphite oxidation-reduction method is commonly used at present due to its large-scale production. The process involves oxidizing graphite with a strong oxidant, followed by the exfoliation to graphene oxide (GO), and then uses a strong reducing agent to reduce GO to graphene. And the preparation of graphene oxide can be achieved by several methods, such as the Brodie, Staudenmaier, Hummers method, or some variation of these methods (Das et al., 2020).

**Figure 3:**
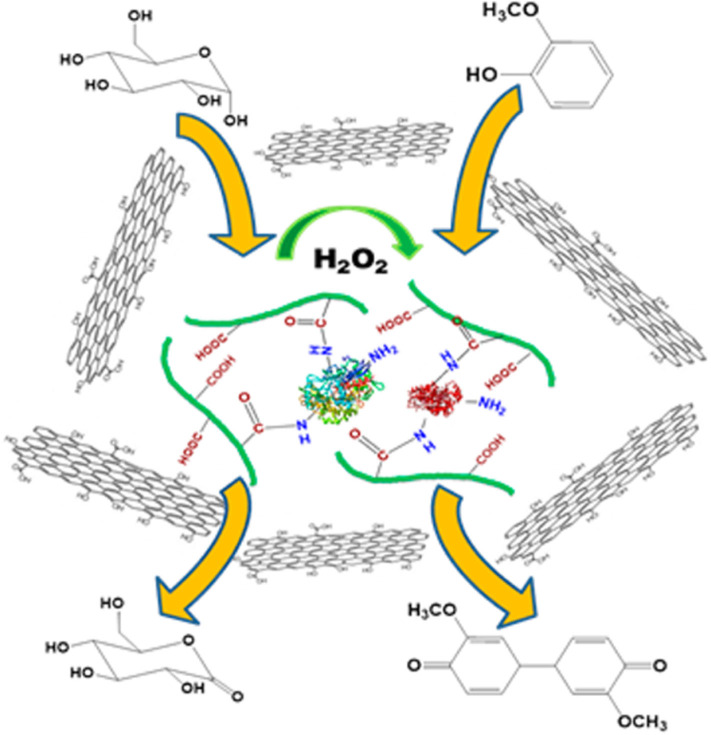
Fabrication of bienzyme-polymer-GO quaternary hybrids for efficient biocatalysis (Zore et al., 2015). Reproduced with permission from ACS publications.

Incredibly large specific surface area, along with the diversity of the functional groups on their surface [such as epoxy (C-O-C), hydroxyl (-OH), carboxyl (-COOH), and carbonyl groups (C-O)], makes graphene and its derivatives ideal substrates for enzyme/multi-enzyme immobilization in biological fields (Table 1 Park and Ruoff, 2009; Zhang et al., 2010; Wang et al., 2011; Ramakrishna et al., 2018). In 2014, Zhao et al. reported the first case of co-immobilization of GOx and glucoamylase onto chemically reduced graphene oxide via non-covalent bonds for one-pot production of gluconic acid from starch (Zhao et al., 2014). The activity and reusability of the enzymes could be improved dramatically by controlling the extent of graphene oxide reduction. In order to keep the GO supported enzymes active at biologically challenging conditions, poly(acrylic acid) was used to covalently conjugate GOx and HRP together before the immobilization of enzymes onto GO (Figure 3; Zore et al., 2015). The resultant bienzyme-polymer-GO quaternary hybrids functioned as active catalysts under extreme pH and high temperature conditions, and the stability of the enzymes was also improved in the presence of a chemical denaturant.

**Table 1:** Examples of multi-enzyme immobilization systems based on graphene and its derivatives.

**Materials**	**Model enzymes**	**Applications**	**Types of multi-immobilization**	**References**
Chemically reduced graphene oxide	GOx and glucoamylase	Preparation of gluconic acid from starch	Random co-immobilization	Zhao et al., 2014
Reduced graphene oxide–dendritic Pd nanoparticles	Cholesterol oxidase and cholesterol esterase	Detection of cholesterol	Random co-immobilization	Dey and Raj, 2014
Graphene oxide	GOx and HRP	Preparation of gluconic acid from glucose	Random co-immobilization	Zore et al., 2015
Chemically reduced graphene oxide	GOx and HRP	Preparation of gluconic acid from glucose	Random co-immobilization	Mathesh et al., 2017
Luminol functionalized graphene oxide	GOx and HRP	Detection of glucose	Random co-immobilization	Li et al., 2018
Hydroxyl and carboxyl modified graphene oxide	Cellulase and GOx	Preparation of gluconic acid from carboxymethyl cellulose	Random co-immobilization	Zhang et al., 2020

Later, Yang's group carried out a systematic investigation to understand the enzyme structural changes and conformations that could enhance activity and substrate channeling of GO-supported enzymes (Mathesh et al., 2017). It was observed that the hydrophobicity of graphene oxides and various enzyme architectures were important attributions for achieving high product conversion rates (GOx and HRP as model enzymes). Randomly immobilized enzymes resulted into extremely efficient substrate channeling with a transient time of close to 0 s, owing to direct molecular channeling of the close to one another enzymes. Inspired from the previous works, a multi-enzyme system for one-pot production of gluconic acid from carboxymethyl cellulose was achieved by co-immobilization of cellulase and GOx on graphene oxide via covalent bonding (Zhang et al., 2020). Moreover (reduced), graphene oxide hybrids can also be used to fabricate multi-enzyme systems for sensitive biosensing (Dey and Raj, 2014; Li et al., 2018).

Graphene and its derivatives are rapidly becoming the most intensively studied carbon-based materials for multi-enzyme immobilization due to their large specific surface area and the diversity of the functional groups. Among graphene and its derivatives, graphene oxide and partially reduced graphene oxide, rather than pristine graphene (with little surface functionalization), are the common-used supports for multi-enzyme immobilization due to the abundant functional groups (Table 1). However, co-immobilized enzymes on water-dispersed graphene oxide are difficult to be separated (Chang et al., 2015), and the partially reduced graphene oxides with strong π-π stacking are easily to aggregate with each other, resulting in the loss of surface functional groups. Moreover, the positions of different enzymes can hardly be precisely controlled on the surface of graphene and its derivatives. Thus, it still remains a long way for graphene and its derivatives to be utilized as support materials for multi-enzyme immobilization in the practical applications.

### Carbon Nanotubes (CNTs)

CNTs are nanoscale circular tubes consisting of a hexagonal arrangement of hybridized carbon atoms, which are formed by a single or multi-layer graphene sheets coiled around the central axis at a certain rotation angle (Ebbesen, 1996). They can be divided into single-walled carbon nanotubes (SWCNTs) and multi-walled carbon nanotubes (MWCNTs). CNTs have received extensive attention due to their extraordinary properties, such as high specific surface area, large aspect ratios, remarkable mechanical strength and excellent chemical stability (Walcarius et al., 2013). There are three main ways to produce nanotubes, including the carbon arc, carbon vapor deposition, and laser ablation of graphite (Dai, 2002).

CNTs are attractive platforms for enzyme/multi-enzyme immobilization (Table 2), in which enzymes can be attached onto CNTs via physical adsorption or cross-linking (Ratautas et al., 2015). CNTs can protect enzymes from inactivation in harsh environments due to the suppression of the lateral interactions between adjacent adsorbed proteins (Asuri et al., 2006). NAD^+^-dependent glycerol dehydrogenase and NAD^+^-regenerating NADH oxidase were co-immobilized on functionalized SWCNTs to achieve cofactor regeneration through the specific interaction between His-tagged enzymes and the modified SWCNTs (Wang et al., 2012). SWCNTs were modified by treating pristine SWCNTs with HNO_3_ and H_2_SO_4_ mixture to produce SWCNT-COOH, and activated with N-hydroxysuccinimide/1-ethyl-3-[3′-(dimethylamino) propyl] carbodiimide. Then N_α_,N_α_-bis (carboxymethyl)-L-lysine hydrate (ANTA)-Co^2+^ was attached to the surface of activated SWCNTs by carbodiimide cross-linking, resulting into SWCNT-ANTA-Co^2+^ complex (Wang et al., 2010, 2012). The resultant nanoscale biocatalysts were employed to produce 4-hydroxy-2-butanone (4H2B) by the oxidation of 1,3-butanediol. The multi-enzyme system was more stable and the yield of 4H2B was almost twice than that of the free enzymes under optimum conditions. For example, SWCNT-GlyDH could still preserve ~90% of its initial activity after 30 min incubation at 60°C (with a half-life of ~400 min), whereas the activity of native GlyDH dropped sharply to ~67% (with a half-life of ~240 min).

**Table 2:** Examples of multi-enzyme immobilization systems based on CNTs.

**Materials**	**Model enzymes**	**Applications**	**Types of multi-immobilization**	**References**
Amino-modified SWCNTs	Glycerol dehydrogenase and NADH oxidase	Preparation of 4-hydroxy-2-butanone from 1,3-butanediol	Random co-immobilization	Wang et al., 2012
MWCNTs	GOx and HRP	Detection of glucose	Random co-immobilization	Huang et al., 2013
MWCNTs	Glucoamylase and GOx	Detection of starch	Random co-immobilization	Lang et al., 2014
MWCNTs	Salicylate hydroxylase and tyrosinase	Detection of methyl salicylate	Random co-immobilization	Fang et al., 2016
CNT Columns	Hydrogenase, NAD^+^ reductase and dehydrogenase	Preparation of L-alanine/(*S*)-1-phenylethanol from pyruvate/acetophenone	Random co-immobilization	Zor et al., 2017

**Figure 4:**
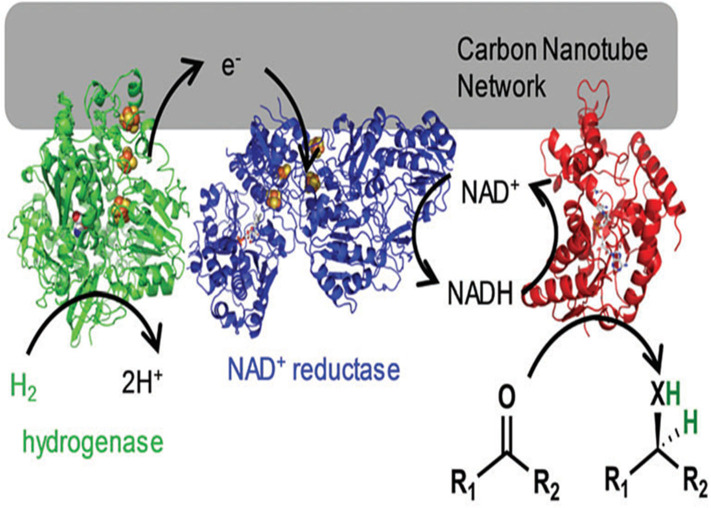
A hydrogenase and NAD^+^ reductase were co-immobilized on the carbon nanotube network for H_2_-driven NADH generation (Zor et al., 2017). Reproduced with permission from The Royal Society of Chemistry.

Similarly, to promote cofactor regeneration, a modular approach to H_2_-driven biocatalytic hydrogenation reactions in continuous flow was presented by Vincent's group (Zor et al., 2017). Hydrogenase, NAD^+^ reductase and alanine dehydrogenase were co-immobilized on carbon nanotube columns, which can act selectively on pyruvate to generate L-alanine (Figure 4). At a low substrate concentration (2 mM), 90% conversion was observed, equating to a total turnover number of 19,600 NADH per NAD+ reductase. When the pyruvate concentration was higher (12.5 mM), 40% conversion could be obtained, equating to a total turnover number of >54,000 NADH per NAD+ reductase. This work showed the possibility of biocatalytic hydrogenations in continuous flow using enzymes immobilized on a CNT-lined quartz column. Furthermore, enzymes co-immobilized on CNTs have also been widely used in electrochemical cascade enzymatic reactions for biosensing (Huang et al., 2013; Lang et al., 2014; Fang et al., 2016).

In recent years, CNTs have received growing attention for multi-enzyme immobilization (Table 2), due to their high specific surface area, large aspect ratios, excellent chemical stability, etc. By modifying various functional groups [such as carboxyl (-COOH), amino (-NH_2_), amino-phenyl (C_6_H_4_NH_2_), benzoic acid (C_6_H_4_COOH), and nitro-phenyl (C_6_H_4_NO_2_)] on the surface of CNTs, the catalytic performances of immobilized enzymes can be improved obviously (Gao and Kyratzis, 2008; Bi et al., 2009; Pang et al., 2010; Yu et al., 2010). Thus, the design of efficient modifiers and the corresponding modification method need to be further investigated for CNTs-based multi-enzyme immobilization.

### Metal-Organic Frameworks (MOFs)

MOFs are porous network structures with tridimensional crystalline, which are constructed by linking metal ions or clusters and organic ligands via coordination bonds (Furukawa et al., 2013; Hu et al., 2018). Geometrical configuration of MOFs (such as linear, octahedron, and plane of conical) is determined by the metal coordination number (Burnett et al., 2012). Since the first preparation of MOFs by cobalt ions and trimesic acid in 1995, MOFs have attracted extensive explorations due to their broad application potentials (Yaghi et al., 1995). To date, solvothermal, microwave-assisted, electrochemical, mechanochemical, and sonochemical methods have been successfully applied for the synthesis of MOFs (Dey et al., 2014). The features of large specific surface area (typically ranging from 1,000 to 10,000 m^2^/g), high porosity (typically ranging from 1 to 10 nm) and adjustable structure indicate that MOFs are ideal supports for enzyme immobilization (Deng et al., 2012; Lian et al., 2017; Liang et al., 2020). In addition, MOFs with large surface-to-volume ratios and multiple enzymes binding sites have been considered as promising carriers for multi-enzyme immobilization (Table 3).

**Table 3:** Examples of multi-enzyme immobilization systems based on MOFs.

**Materials**	**Model enzymes**	**Applications**	**Types of multi-immobilization**	**References**
ZIF-8	GOx and HRP	Detection of glucose	Random co-immobilization	Wu et al., 2015
PCN-888	GOx and HRP	Preparation of gluconic acid from glucose	Positional co-immobilization	Lian et al., 2016
HKUST-1@Fe_3_O_4_ nanoparticles	GOx and HRP	Preparation of gluconic acid from glucose	Compartmentalization/Random co-immobilization	Chen et al., 2017
ZIF-8	Glucoamylase and α-amylase	Preparation of glucose from starch	Random co-immobilization	Salgaonkar et al., 2018
PCN-333	Cholesterol oxidase and HRP	Detection of cholesterol	Compartmentalization	Zhao et al., 2019
Amine-functionalized MIL-101(Cr)	Carbonic anhydrase, formate dehydrogenase and glutamate dehydrogenase	Preparation of formic acid from CO_2_	Compartmentalization	Li et al., 2019

Ge's groups reported the first case of a multiple enzyme-incorporated MOF via a one-step and facile synthesis procedure in 2015 (Wu et al., 2015). The multi-enzyme system was constructed by embedding GOx and HRP in zeolitic imidazolate framework (ZIF-8) via co-precipitation in aqueous solution at ambient conditions, which exhibited high sensitivity (limit of glucose detection is 0.5 mM), high selectivity (no obvious activity toward other interfering compounds including fructose, mannose, galactose, maltose, lactose, and albumin) and long-term storage stability (~80% retention of initial overall activity after 7 days). Later, the above *in situ* entrapment method was adopted to co-immobilize α-amylase and glucoamylase in ZIF-8 for one-pot starch hydrolysis by another research group (Salgaonkar et al., 2018). More recently, Ge's groups developed a coarse-grained, particle-based model to understand the origin of high efficiency in confined multi-enzyme catalysis. They found that the property of reaction intermediates was the key in determining the reaction kinetics (Cao et al., 2019).

**Figure 5:**
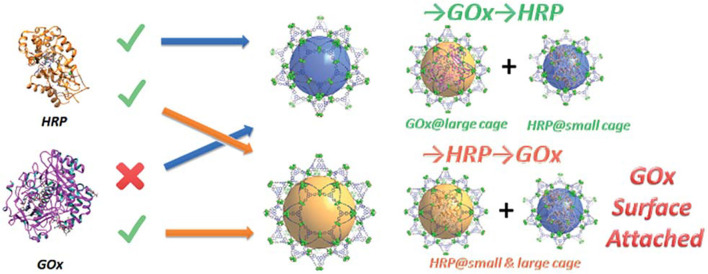
Graphic representation of the stepwise encapsulation of GOx and HRP with different orders (Lian et al., 2016). Reproduced with permission from The Royal Society of Chemistry.

To precisely control the distribution of GOx and HRP, Zhou's group rationally designed and synthesized a novel hierarchical mesoporous MOF PCN-888 with three types of cavity (Lian et al., 2016). The largest cavity (6.2 nm) and the intermediate cavity (5.0 nm) could only accommodate one molecule of GOx and HRP, repectively (Figure 5). The smallest cavity (2.0 nm) was left empty as a diffusion pathway for substrates and products, which had sufficient size for neither GOx nor HRP. The resultant multi-enzyme system exhibited high catalytic efficiency (*k*_cat_ = 2.411 × 10^4^ s^−1^, *K*_m_ = 9.67 mM, and *v*_max_ = 1.96 × 10^−3^ mM s^−1^), good cycling performance (the activity remained almost the same within four catalytic cycles) as well as the protective effect of the immobilized enzymes against trypsin digestion. Similarly, cholesterol oxidase and HRP were co-immobilized in the mesoporous cages and the surface of MOF PCN-333(Al), which was used as a colorimetric biosensor for the detection of cholesterol (Zhao et al., 2019). Tan and Lv's group also have made the contribution in the area of MOFs (HKUST-1) for multi-enzyme immobilization (Chen et al., 2017; Li et al., 2019). They adopted a layer-by-layer self-assembly approach to achieve the sequential co-immobilization of multi-enzymes using MOFs in layered structure as the solid scaffold, which were used for the efficient biocatalytic conversion of adsorbed CO_2_ into formate.

Recently, MOFs have attracted considerable scientific interests as enzymes co-immobilized supports due to the large surface-to-volume ratios and multiple enzyme binding sites, in which ZIF-8, PCN-888, HKUST-1, etc. are commonly used (Table 3). In spite of their advantages, the problems associated with the long-term water stability and potential leaching of toxic metal ions still need to be addressed to achieve the ideal multi-enzyme-MOF composites, which will be in line with the concept of sustainable green production. The future of MOFs for multi-enzyme immobilization lies in the simple, gentle, and eco-friendly synthesis method along with their potential industrial applications, in which the compatibility of the synthetic conditions with fragile enzymes should also be taken into consideration.

### DNA Nanostructures

DNA nanotechnology has been demonstrated as a reliable way to fabricate complex biomolecular nanostructures due to the programmability of DNA hybridization (Pinheiro et al., 2011). For multi-enzyme immobilization, it is valuable to reduce the mass transfer resistance by controlling the relative positions and directions of different enzymes in a confined space. Thus, DNA nanotechnology has been utilized as an effective tool for the co-immobilization of various enzymes (Table 4), in which spatially addressable DNA nanostructures can facilitate the precise self-assembly of different enzymes to improve the substrate channeling.

**Table 4:** Examples of multi-enzyme immobilization systems based on DNA nanostructures.

**Materials**	**Model enzymes**	**Applications**	**Types of multi-immobilization**	**References**
DNA origami (hexagon-like strips)	GOx and HRP	Preparation of gluconic acid from glucose	Positional co-immobilization	Wilner et al., 2009
Rectangular DNA origami	GOx and HRP	Preparation of gluconic acid from glucose	Positional co-immobilization	Fu et al., 2012
DNA-inhibitor scaffold	Invertase, GOx and HRP	Preparation of O-dianisidine from sucrose and glucose	Positional co-immobilization	Liu et al., 2013
Rectangular DNA origami	GOx and HRP	Preparation of gluconic acid from glucose	Positional co-immobilization	Fu et al., 2013
DNA origami	G6pDH and MDH	Preparation of malic acid from oxaloacetic acid	Positional co-immobilization	Fu et al., 2014
DNA hybrid (polystyrene)	GOx and HRP	Preparation of gluconic acid from glucose	Positional co-immobilization	Jia et al., 2015
Rectangular DNA origami	Xylose reductase and xylitol dehydrogenase	Preparation of xylulose from D-xylose	Positional co-immobilization	Ngo et al., 2016
Triangle DNA origami	GOx and Catalase	Preparation of gluconic acid from glucose	Compartmentalization	Sun et al., 2017
DNA hybrid (dopamine@ magnetic nanoparticles)	GOx and HRP	Preparation of gluconic acid from glucose	Random co-immobilization	Yang et al., 2017
DNA hybrid (Fe_3_O_4_@SiO_2_ nanoparticles)	GOx and HRP	Detection of target DNA	Random co-immobilization	Song et al., 2018
DNA nanotweezer	GOx and HRP	Detection of glucose	Positional co-immobilization	Kou et al., 2018
Rectangular DNA origami	Glucose 6-phosphate dehydrogenase and lactate dehydrogenase	Preparation of 6-Phosphogluconic acid, lactate from glucose-6-phosphate, pyruvate	Positional co-immobilization	Chen Y. et al., 2018
Triangle DNA origami	Amylase, maltase and glucokinase	Preparation of glucose-6-phosphate from maltoheptaose	Positional co-immobilization	Klein et al., 2019
DNA Tetrahedron scaffold	GOx and HRP	Detection of glucose	Positional co-immobilization	Wang D. et al., 2019

DNA origami refers to the self-assembly of highly complex nanostructures by combining a long single strand of DNA with a series of short and engineered DNA fragments via the specific interaction between complementary base pairs (Rothemund, 2006). Immobilization of enzymes onto the DNA scaffold with precise positions is a promising approach for the self-organization of enzyme-DNA origami composite nanostructures. In 2009, Willner's group first explored the properties of the self-assembled DNA nanostructures as co-immobilized scaffolds for GOx and HRP (Wilner et al., 2009). The biocatalytic cascade reaction was activated by tethering GOx and HRP on two-hexagon and four-hexagon DNA strips. The results showed that the biocatalytic rate operating on the two-hexagon templates was about 20 times faster than that on four-hexagon templates, owing to the high local concentration of the intermediate H_2_O_2_.

**Figure 6:**
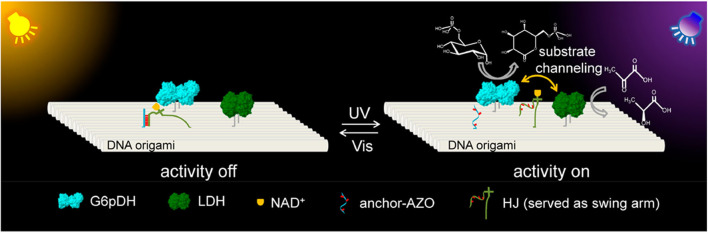
G6pDH-LDH enzyme cascade and NAD^+^-HJ swing arm assembly on DNA origami scaffold (Chen Y. et al., 2018). The swing arm was fixed to be far away from the two enzymes under Vis due to the hybridization of HJ-arm-AZO and anchor-AZO, which turned off enzyme cascade activity. Under UV light, the swing arm was released to freely swing between both enzymes to switch on the enzyme cascade activity. Reproduced with permission from ACS publications.

Yan's group systematically studied the activity of a GOx/HRP cascade spatially organized on DNA origami tiles as a function of interenzyme spacing and position (with the interenzyme distances varied from 10 to 65 nm) (Fu et al., 2012). It was observed that closely spaced enzymes (10 nm apart) showed strongly enhanced activity and the activity dropped dramatically for enzymes as little as 20 nm apart. They also precisely mediated glucose-6-phosphate dehydrogenase (G6pDH) and malic dehydrogenase (MDH) with an artificial swinging arm, which showed that enzymatic activity was closely related to the number, position, and direction of the swinging arm relative to the enzymes (Fu et al., 2014). More recently, Yan's and Yang's groups co-developed a synthetic light-driven substrate channeling system based on DNA origami (Figure 6), which could precisely regulate the enzyme cascade activity (Chen Y. et al., 2018). Glucose-6-phosphate dehydrogenase (G6pDH) and lactate dehydrogenase (LDH) were served as the model cascade enzymes, and nicotinamide adenine dinucleotide (NAD^+^) was served as the cofactor, which were conjugated with a Holliday junction (HJ) to form a swing arm between G6pDH and LDH on DNA origami. Under visible light (Vis) irradiation, azobenzene-modified arm of HJ (called HJ-arm-AZO) hybridized with the azobenzene-modified anchor strand (called anchor-AZO), leading the swing arm far away from the enzyme cascade, which switched off its activity (left panel in Figure 6). When ultraviolet light (UV) was applied, the isomerization of azobenzene resulted in the dehybridization of HJ-arm-AZO and anchor-AZO, and the swing arm was released to swing between the two enzymes, thus strongly enhancing the cascade activity (right panel in Figure 6).

Lu's group demonstrated a general design of robust enzyme nanocomplex by conjugating invertase, GOx, and HRP via the specific binding of DNA-inhibitor scaffold and multiple enzymes, followed by the encapsulation in a thin polymer shell (Liu et al., 2013). The enzyme nanocomplex showed improved efficiency and enhanced stability as well as complementary and synergic functions. The method was also applied for the design of an alcohol prophylactic and antidote by assembling alcohol oxidase (AOx) and catalase on the DNA scaffold. Ngo et al. reported the fabrication of an artificial enzyme cascade based on the xylose metabolic pathway, in which xylose reductase and xylitol dehydrogenase were localized to specific positions on DNA origami with three rectangular cavities by DNA-binding protein adaptors (Ngo et al., 2016). In addition to triangle DNA origami, DNA tetrahedron scaffold were also designed and used for the site-specific co-immobilization of various enzymes (Sun et al., 2017; Klein et al., 2019; Wang D. et al., 2019).

DNA tweezer is a regenerated scaffold that interenzyme distance can be easily manipulated. When external triggers exist, such as light or metal ions, DNA tweezers could be achieved autonomous switchable motion with an addressable conformation change. A regenerated DNA tweezer was designed to regulate interenzyme spacing via “open-close-open” strategy for highly efficient enzyme cascade amplification (Kou et al., 2018), which can overcome the drawbacks of inflexible, time-consuming operation of DNA origami method. GOx and HRP were, respectively, modified as model enzymes with the arms of opened DNA tweezer, which can enhance the catalytic efficiency for sensitive target DNA analysis at a low detection limit down to 30 fM.

Apart from DNA origami structures and DNA tweezers, DNA hybrids have also been utilized for multi-enzyme immobilization (Jia et al., 2015; Yang et al., 2017; Song et al., 2018). For example, Yang's group fabricated a multi-enzyme system by co-immobilizing GOx and HRP on dopamine functionalized magnetic nanoparticles through DNA directed immobilization. The resultant multi-enzyme system exhibited precise enzyme ratio control, high catalytic efficiency, magnetic recyclability, and enhanced stability. The Michaelis constant *K*_m_ and specificity constant (*k*_cat_/*K*_m_) of the multi-enzyme system were 1.41 mM and 5.02 s^−1^ mM^−1^, respectively, which were approximately twice the corresponding values of the free enzymes.

Over the last few years, biocompatible DNA nanostructures with tight coding and paring between bases have received considerable attentions in the field of biotechnology. And DNA-based materials have been proved as a reliable platform for the fabrication of multi-enzyme systems with controlled spatial arrangements and precise positions (Table 4), which can improve enzyme cascade catalytic efficiency via substrate channeling. However, due to the operation difficulty and high cost, this promising technology can hardly meet the requirements of large-scale industry applications in the current stage. With the development of DNA nanotechnology, it is believed that the problems will be solved and DNA nanostructures will gradually enter the practical applications in the near future.

### Polymers

Polymers with flexibility and diversity can be rationally designed based on the enzyme characteristics, which have been proved as one of the most promising materials for enzyme immobilization. They have several beneficial properties, such as good colloidal and mechanical stability, tailored permeability, and stimuli responsiveness (Bermudez et al., 2002; Du and O'Reilly, 2009; Jochems et al., 2011). It has been demonstrated that polymers are one of the common-used traditional carriers for multi-enzyme immobilization and have been studied for a long time. For example, Liu et al. reported an amperometric biosensor for the detection of glucose and lactose via the co-immobilization of GOD, β-alactosidase, mutarotase in β-cyclodextrin polymer, and the three enzymes were cross-linked by the polymer (Liu et al., 1998). Herein, a series of recently reported polymer-based materials for enzymes co-immobilization will be introduced (Table 5), including polymersomes, polymer nanostructures, polymer films, etc.

**Table 5:** Examples of multi-enzyme immobilization systems based on polymers.

**Materials**	**Model enzymes**	**Applications**	**Types of multi-immobilization**	**References**
Polystyrene nanoparticles	FDH, FaldDH, ADH and GDH	Preparation of methanol from CO_2_	Random co-immobilization	El-Zahab et al., 2008
Polydopamine microcapsule	α-amylase, β-amylase and glucosidase	Preparation of isomaltooligosaccharide from starch	Compartmentalization	Zhang et al., 2011
Dendronized polymer (de-PG1_2000_)	Superoxide dismutase and HRP	Preparation of ABTS^∙−^, O_2_ from ABTS^2−^, ${\text{O}}_{2}^{∙-}$	Random co-immobilization	Grotzky et al., 2012
Polyurethane hollow nanofiber	3a-HSD, DP and NADH	Preparation of 3-O-bile acid from bile acids	Random co-immobilization	Ji et al., 2014
ABA polymersomes	GOx and HRP	Preparation of D-gluconolactone from glucose	Compartmentalization	Siti et al., 2014
Supramolecular-polymeric hybrid hydrogel	GOx and HRP	Preparation of gluconic acid, phenazine-2,3-diamine from glucose, O-phenylenediamine	Random co-immobilization	Wei et al., 2016
PNIPAm-PEI	Pyruvate kinase and L-lactic dehydrogenase	Preparation of lactate from phosphoenol pyruvate	Random co-immobilization/compartmentalization	Dubey et al., 2016
PMOXA-PDMS-PMOXA Polymersomes	AGE, NAL and CSS	Preparation of CMP-Neu5Ac from GlcNAc, pyruvate and CTP	Compartmentalization	Klermund et al., 2017
Polyglycidyl methacrylate sphere	GOx and catalase	Preparation of gluconic acid from glucose	Random co-immobilization	Liao et al., 2019
LDPE-g-poly(PEGDA)/BG-g-PAANa film	β-glucosidase and cellulase	Preparation of glucose from cellulose	Compartmentalization	Wang Y. et al., 2019

**Figure 7:**
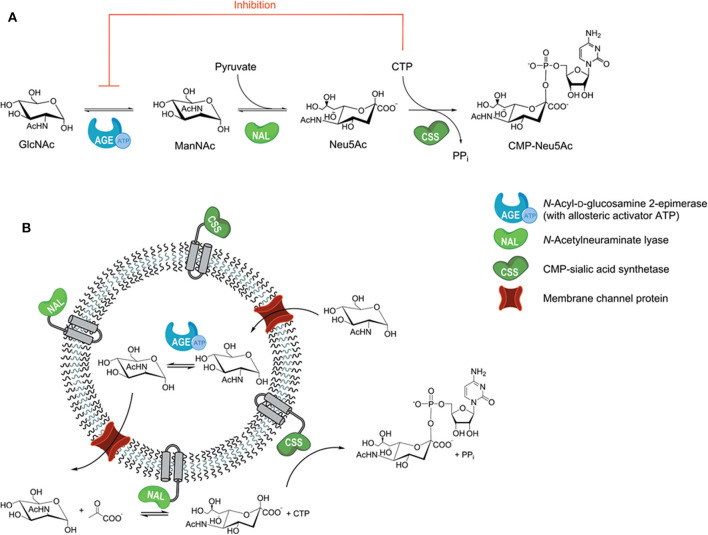
**(A)** Three-step preparation of CMP-Neu5Ac; **(B)** compartmentalized reaction scheme in polymersomes (Klermund et al., 2017). Reproduced with permission from ACS publications.

Polymersomes (i.e., polymer-based liposomes) are self-assembled polymer shells composed of block copolymer amphiphiles (Discher et al., 1999), in which multiple enzymes can be blocked in the polymer vesicles. Polymersomes with reconstituted channel proteins can be designed rationally to be more suitable for multi-enzyme immobilization, which will improve the mass transfer efficiency of the cascade reaction. For example, a channel-equipped polymersome-in-polymersome architecture has been constructed for multi-enzyme biocatalysis, where the passive protein channel (outer-membrane protein F) was embedded into the inner membrane to allow for molecule exchange (Siti et al., 2014). A porin-functionalized polymersome was prepared to avoid the incompatible reactions by compartmentalization, which served as a naturally selective barrier between substrates and inhibitors (Figure 7; Klermund et al., 2017). To separate the incompatible N-acyl-D-glucosamine 2-epimerase (AGE) and CMP-sialic acid synthetase (CSS) reactions, the AGE was encapsulated in the lumen of poly(methyloxazoline)_15_-poly(dimethylsiloxane)_68_-poly(methyloxazoline)_15_ (PMOXA-PDMS-PMOXA) polymersomes (Figure 7B) during vesicle formation and nacetylneuraminate lyase (NAL) and the CSS were co-immobilized on the outer surface using hydrophobic peptide anchors. The three-step preparation (Figure 7A) of CMP-N-acetylneuraminic acid (CMP-Neu5Ac) from N-acetylglucosamine (GlcNAc), pyruvate and cytidine triphosphate (CTP) was improved 2.2-fold compared to the free enzymes.

Polymer nanostructures (such as nanoparticles and nanofibers) have also been used as carriers for the fabrication of multi-enzyme systems. Wang's group prepared polystyrene particles to co-immobilize formate dehydrogenase (FDH), formaldehyde dehydrogenase (FaldDH), alcohol dehydrogenase (ADH), glutamate dehydrogenase (GDH), and cofactor NADH/NAD^+^, which achieved efficient cofactor regeneration for the synthesis of methanol from CO_2_ (El-Zahab et al., 2008). Similarly, polyglycidyl methacrylate (PGMA) spheres were also used for the co-immobilization of GOx and catalase for the preparation of gluconic acid from glucose (Liao et al., 2019). Zhang's group fabricated a polyurethane hollow nanofiber via a co-axial electrospinning technique, which could be used for the co-immobilization of 3a-hydroxysteroid dehydrogenase (3a-HSD), diaphorase (DP), and NADH (Ji et al., 2014). In this multi-enzyme system, bile acids was oxidized and 2,6-dichlorophenolindophenol was reduced with cofactor regeneration, which presented 75% recovery activity and more than a 170-fold increase in half-life at 25°C.

A suitable polymer film was also exploited to co-immobilize β-glucosidase (BG) and cellulose for the production of glucose from cellulose, which was formed by visible-light-induced graft polymerization (Wang Y. et al., 2019). The layered polyethylene (LDPE) film consisted of a thin PEG hydrogel (as the inner layer) and a sodium polyacrylate (PAANa) brush (as the outer layer), where BG/cellulose was immobilized in/on the inner/outer layer, respectively. The resultant dual-enzyme system exhibited 82 and 20% increase in catalytic activity, compared with the single cellulase system and isolated BG/cellulase immobilized system, respectively. Moreover, 93% of carboxymethylcellulose sodium salt (CMC) activity could be maintained after 10 cycles of hydrolysis, exhibiting excellent reusability.

Moreover, the dendronized polymers (Grotzky et al., 2012), polydopamine microcapsules (Zhang et al., 2011), and polymer hybrid hydrogels (Wei et al., 2016) have also been synthesized for the multi-enzyme immobilization. For example, hybrid hydrogels were fabricated by dual enzyme (GOx and HRP) mediated redox initiation polymerization, which combined the merits of higher mechanical strength and porous networks (Wei et al., 2016). The immobilized GOx/HRP in the hybrid hydrogels revealed super activity, as indicated by the highest *k*_cat_ value (7.348 s^−1^), which is approximately 1.16 ± 0.05 times higher than that of the free GOx/HRP (6.340 s^−1^).

In the past several decades, it has been demonstrated that polymer-based materials are ideal support materials for multi-enzyme immobilization due to their good mechanical strength, easily adjustable morphologies and stable properties (Table 5). However, some limitations/problems still existed in the polymers based multi-enzyme immobilization systems, including the unprecise arrangement of different enzymes and the loss of chemical groups from the carriers. Even though, polymer-based materials are still one of the most common-used traditional supports for multi-enzyme immobilization in the industrial/practical applications.

### Silica

Silica (SiO_2_) materials are one of the widely used mesoporous structures with various applications (Zhou and Hartmann, 2013), which possess ordered pore structure, narrow pore size distribution, large specific surface area (~1,000 m^2^ g^−1^) and high stability. Thus, mesoporous silica have attracted reasonable attention as the enzyme immobilization supports (Magner, 2013), and their unique features also suggest that they are ideal supports for multi-enzyme immobilization. For example, Van Aken et al. reported the case of co-immobilization of manganese peroxidase and glucose oxidase on porous aminoalkylethoxysilane activated silica beads by glutaraldehyde assisted covalent bindings, which was used for the production of gluconic acid from glucose (Van Aken et al., 2000). Silica materials are one of the traditional enzyme co-immobilization carriers and have been studied for a long time (Cho et al., 2012). Herein, some recent important works for multi-enzyme immobilization based on SiO_2_ carriers with different morphology will be provided (Table 6), including microcapsules, colloidosomes, core-shell nanoparticles etc.

**Table 6:** Examples of multi-enzyme immobilization systems based on silica.

**Materials**	**Model enzymes**	**Applications**	**Types of multi-immobilization**	**References**
Aminoalkylethoxysilane activated silica beads	GOx and HRP	Preparation of gluconic acid from glucose	Random co-immobilization	Van Aken et al., 2000
Catechol-modified gelatin-silica microcapsules	FateDH, FaldDH and YADH	Preparation of methanol from CO_2_	Compartmentalization	Wang et al., 2014
Nanoscale silica layers	Cellulase, glucokinase and phosphoglucose dehydrogenase	Preparation of cellobiose from 6-phosphogluconolactone	Compartmentalization	Begum et al., 2015
Poly(acrylic acid) brushes-nano spherical silica	GOx and HRP	Detection of glucose	Random co-immobilization	Zhao et al., 2016
SiO_2_ nanoparticle colloidosomes	GOx and lignin peroxidase	Preparation of ethyl acetate from pyridine-N-oxide	Compartmentalization	Liu et al., 2018
Core-shell magnetic SiO_2_ hybrid nanoparticles	Cellulase and lysozyme	Biocatalysis (cell-walls degradation)	Random co-immobilization	Chen Q. et al., 2018
Amino-modified Fe_3_O_4_/SiO_2_ core-shell nanospheres	Glucoamylase and α-amylase	Preparation of glucose from starch	Random co-immobilization	Bian et al., 2018

A ultrathin, hybrid silica microcapsule was designed to construct an efficient multi-enzyme system for the production of methanol from CO_2_ (Wang et al., 2014), which was prepared through the deposition of catechol-modified gelatin, followed by *in situ* growth of silica nanoparticles on the gelatin layer. Formate dehydrogenase (FateDH), formaldehyde dehydrogenase (FaldDH), and alcohol dehydrogenase (YADH) were immobilized through physical entrapment in the microcapsule lumen, covalent attachment onto the catechol-modified gelatin layer, and physical entrapment in the silica layer, respectively. The ordered assembly of enzymes and the adjustable pore sizes of the scaffold facilitated the direct transfer of an intermediate between consecutive enzymes, resulting remarkably higher yield (71.6%) and selectivity (86.7%) of catalysis systems than that of free enzymes (35.5% yield and 47.3% selectivity). Moreover, this multi-enzyme co-immobilized system exhibited good recyclability with 52.6% yield retention after 9 recycles. Similarly, nanoscale silica layers coated diatom silica microparticles were designed for the fabrication of an artificial enzyme cascade system, in which three hexaarginine-tagged enzymes (i.e., cellulase, glucokinase, and phosphoglucose dehydrogenase) were co-immobilized in the five layers of silica via peptide-mediated layer-by-layer mineralization (Begum et al., 2015). Moreover, a poly(acrylic acid) brushes-nanospherical silica was also used as co-immobilized support for the construction of a bienzymatic biosensor for glucose detection (Zhao et al., 2016).

**Figure 8:**
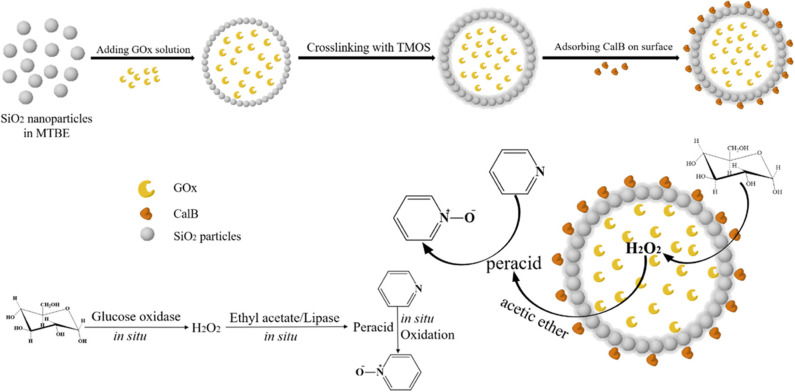
Schematic illustration of the fabrication of the dual-enzyme colloidosome reactor for biphasic catalysis of pyridine (MTBE is *tert*-butyl methyl ether and TMOS is tetramethyl orthosilicate) (Liu et al., 2018). Reproduced with permission from ACS publications.

A colloidosome composed of SiO_2_ nanoparticles was synthesized as the microreactor for dualenzyme cascade biphasic reaction (Figure 8), in which GOx was compartmentalized inside the colloidosome and *Candida antarctica* lipase (CalB) was adsorbed on the outer surfaces of the colloidosome (Liu et al., 2018). Glucose, as the energy source, was catalyzed by GOx to produce hydrogen peroxide (H_2_O_2_) and H_2_O_2_ diffused out of the microcapsules was employed by CalB to catalyze the perhydrolysis of ethyl acetate. The generated peracids could oxidize of N-heteroaromatic compounds *in situ*. The bioinspired dual-enzyme amphiphilic colloidosome reactor showed high biphasic catalytic performance of pyridine, and no obvious yield decline was observed in the four cycles.

Core-shell magnetic silica hybrid nanoparticles have also been designed through coating silica shell on the surface of magnetic core, which exhibited large specific surface area and easy recovery (Chen Q. et al., 2018). Cellulase and lysozyme were co-immobilized on the surface of amino-functionalized magnetic nanoparticles, which showed greater thermal stability and wider pH tolerance than free enzymes under harsh conditions. Similarly, α-amylase and glucoamylase were covalently co-immobilized on amino-modified Fe_3_O_4_/SiO_2_ core-shell nanospheres with a Fe^3+^-tannic acid film for one-pot starch hydrolysis (Bian et al., 2018).

In the past several decades, it has been demonstrated that silica-based materials are ideal supports for multi-enzyme immobilization (Table 6), due to their ordered pore structures, narrow pore size distributions and large specific surface areas. However, some limitations/problems still exist in the silica based multi-enzyme immobilization systems, including the easy enzymes leaching and unprecise arrangement of different enzymes. A major development direction may be the formation of composites with other materials (such as MOF and DNA), which will improve the immobilized efficiency or substrate channeling of silica-based multi-enzyme systems.

### Others

In addition to the above 6 main support materials, other carriers (such as metal, TiO_2_ and natural polymers) have also been used for the fabrication of efficient multi-enzyme systems (Table 7).

**Table 7:** Examples of multi-enzyme immobilization systems based on other materials.

**Materials**	**Model enzymes**	**Applications**	**Types of multi-immobilization**	**References**
Nanoporous gold	GOx and lignin peroxidase	Preparation of gluconic acid from glucose	Random co-immobilization	Qiu et al., 2009
Amino-modified silver dendritic hierarchical nanostructure	GOx and glucoamylase	Preparation of gluconic acid from starch	Random co-immobilization	Rezaei et al., 2019
OilGOxopa-functionalized TiO_2_ nanoparticles	Formate dehydrogenase and formaldehyde dehydrogenase	Preparation of formaldehyde from CO_2_	Compartmentalization	Shi et al., 2012
PDA-PEI/TiO_2_ hybrid microcapsules	GOx, catalase	Preparation of gluconic acid from glucose	Random co-immobilization	Shi et al., 2015
Agarose beads modified by glyoxyl and boronate	NADH oxidase, formate dehydrogenase and peroxidase	Preparation of insoluble phenolic derivatives from aromatic compound (phenol, 2,4-dichlorophenol, 4-aminophenol or α-naphthol), formic acid	Random co-immobilization	Rocha-Martin et al., 2014
Octyl-modified agarose	Lipase and β-galactosidase	Preparation of galactose from triacetin and lactose	Compartmentalization	Peirce et al., 2016

Porous metal nanoparticles (e.g., gold and silver) have been used as co-immobilized carriers for different enzymes. Lignin peroxidase and GOx were co-immobilized on the surface of nanoporous gold via physical adsorption, which exhibited good potentials in biocatalysis and biosensing (Qiu et al., 2009). GOx and glucoamylase were covalently attached to the amino modified dendritic Ag hierarchical nanostructure for the efficient one-pot conversion of starch into gluconic acid (Rezaei et al., 2019), and the resultant multi-enzyme system exhibited high total activity and reusability over a wide range of pH and temperature values.

TiO_2_ based structures (e.g., TiO_2_ nanoparticles and TiO_2_ hybrid microcapsules) have also been utilized as support materials for multi-enzyme immobilization. A separated multi-enzyme system based on TiO_2_ nanoparticles was constructed for converting CO_2_ to formaldehyde, in which formate dehydrogenase was entrapped in the TiO_2_ nanoparticles during the material formation and formaldehyde dehydrogenase was immobilized on the surface (Shi et al., 2012). It was found that smaller nanoparticle-based multi-enzyme system displayed higher specific activity, yield and selectivity, while larger ones exhibited superior recycling stability. Similarly, catalase and GOx were encapsulated in the polydopamine-polyethylenimine (PDA-PEI)/TiO_2_ hybrid microcapsules for efficient biocatalysis and biosensing (Shi et al., 2015).

Due to the good biocompatibility, low price and biodegradability, natural polymers (e.g., agarose) have been used as multi-enzyme immobilized supports for decades (Liu et al., 2002; Shao et al., 2002). Recently, formate dehydrogenase and NADH-oxidase were co-immobilized onto glyoxyl activated agarose beads and peroxidase was immobilized onto the boronate activated agarose for the fabrication of the multi-enzyme biocatalyst (Rocha-Martin et al., 2014), which can oxidize phenols using oxygen and formic acid as substrates without undesired by-products. A hetero-functional octyl-agarose support for multi-enzyme immobilization was also developed for the preparation of galactose from triacetin and lactose, in which lipase and β-galactosidase were co-immobilized on the hydrophobic surface of the supports via different immobilization strategies (Peirce et al., 2016).

## Conclusion

In this review, a series of recent support materials and their corresponding attachment techniques for multi-enzyme immobilization have been presented. Six main catalogs of supports for the fabrication of multi-enzyme systems have been overviewed, including graphene and its derivatives, CNTs, MOFs, DNA nanostructures, polymers, and silica. In addition, three main techniques for multi-enzyme immobilization have also been discussed, including random co-immobilization, positional co-immobilization, and compartmentalization. By the mimic of multiple enzyme arrangement in cells *in vivo*, co-immobilized enzymes can catalyze raw materials to synthesize many valuable products *in vitro*, such as pharmaceuticals, cosmetics, and nutrition. Support materials not only can serve as scaffold, but also regulate the catalytic properties of enzymes, which will increase the enzymatic activity via substrate channeling and improve the stability and reusability of enzymes. However, co-immobilized multi-enzyme biocatalysis still faces some problems, such as precisely controlling the synthetic substrate channeling, lowering the overall production costs, developing greener and more biocompatible supports. In the future, the design of a co-immobilized multi-enzyme catalysis system with high catalytic efficiency, reusability, and practical operations will be the research priority in the fields of biotechnology and bioengineering.

## Author Contributions

KX wrote and revised the manuscript under the guidance of RZ and YZ. XC helped to draw the figures and write the manuscript. All authors contributed to the article and approved the submitted version.

## Conflict of Interest

The authors declare that the research was conducted in the absence of any commercial or financial relationships that could be construed as a potential conflict of interest.
